# An adaptive optimized handover decision model for heterogeneous networks

**DOI:** 10.1371/journal.pone.0294411

**Published:** 2023-11-15

**Authors:** Nada Ahmed Ezz-Eldien, Heba M. Abdel-Atty, Mahmoud I. Abdalla, Korany R. Mahmoud, Mohamed F. Abdelkader

**Affiliations:** 1 Electrical Engineering Department, Faculty of Engineering, Port Said University, Port Said, Egypt; 2 Electronics & Communications Engineering Department, Faculty of Engineering, Zagazig University, Zagazig, Egypt; 3 Electrical Engineering Department, Faculty of Engineering, October 6 University, 6^th^ of October, Egypt; 4 Department of Electronics and Communications, Faculty of Engineering, Helwan University, Cairo, Egypt; 5 National Telecommunications Regulatory Authority, Ministry of Communication and Information Technology, Giza, Egypt; Jaramogi Oginga Odinga University of Science and Technology, KENYA

## Abstract

A heterogeneous network (HetNet), combining different technologies, is considered a promising solution adopted by several upcoming generations of mobile networks to keep up with the rapid development of mobile users’ requirements while improving network performance. In this scenario, a vertical handover (VHO) algorithm is responsible for ensuring the continuity of the ongoing user connection while moving within the coverage of the HetNet. Although various VHO algorithms were proposed, achieving efficient performance from both network and user perspectives remains challenging. This paper proposes an adaptive optimized vertical handover algorithm based on a multi-attribute decision-making (MADM) algorithm integrated with particle swarm optimization and gravitational search algorithm (PSOGSA) as a framework to implement the handover process. The algorithm includes three main ideas. Firstly, a network selection framework is proposed considering the most important criteria, including signal strength and other networks’ attributes, along with users’ characteristics regarding their mobility and service preferences. Secondly, two new parameters are introduced as control handover parameters named load factor (LF) and score priority (SP) to reduce unnecessary handovers and the overall HetNet power consumption while achieving balanced load distribution. Lastly, the desired aims are formulated as an objective function, then the PSOGSA algorithm is used to reach the optimal values of both LF and SP, which will be considered when executing the handover algorithm. The presented algorithm is simulated in a heterogeneous wireless network where the fifth-generation (5G) wireless technology coexists with other radio access networks to improve the evaluation field of the proposed algorithm. Also, the proposed algorithm’s performance is evaluated in the case of using various MADM algorithms. The simulation results show that the proposed adaptive optimized approach attains efficient performance by decreasing unnecessary handovers by more than 40% and achieving much better load distribution by around 20% to 40%, outperforming traditional handover approaches.

## Introduction

The upcoming mobile network generations are in a constant quest to fulfill the requested performance and achieve the service quality demanded by various users of different application types in all fields [1, 2]. Integrating various network access with different properties and abilities to provide many services in a heterogeneous network (HetNet) is considered a committed way to make the best benefits of all existing wireless technologies [3]. The goal is to transfer the running connection to a more suitable network whenever needed. To achieve that, there is an essential need for a well-organized handover algorithm between the various candidate networks available for connection establishment [4, 5].

Many developed methods have characterized the handover process for the past decade based on the network selection criteria. These methods can be broadly categorized into single-criterion and multiple-criteria-based algorithms [6–8]. Single criterion methods, such as relying on each candidate network’s received signal strength (RSS), provide simplicity but lack sufficient accuracy. On the other hand, multiple criteria approaches, such as multi-attribute decision-making (MADM) techniques, require higher complexity but achieve much more desirable reliability. For example, they require full knowledge of the concerned attributes for all candidate networks gathering them in one score function to reach the correct handed-to network as the one with the highest score [9].

Many MADM algorithms have been proposed, including Technique for Order Preference by Similarity to Ideal Solution (TOPSIS), Weighted Product Model (WPM), Simple Additive Weighting (SAW), and Analytic Hierarchy Process (AHP). The first three algorithms follow the same procedure to reach the final decision: weighting the candidates’ attributes and then gathering the weighted attributes in a score function. The AHP method, introduced by Saaty in 1980, can be considered an efficient method for making decisions by comparing candidates for each criterion to find the preference for one alternative over the others [10, 11]. To enclose user satisfaction with interested attributes, some researchers have adopted utility theory to represent network attributes, as in [12].

To highlight the proposed algorithm’s effort, some related studies are mentioned next. In [13], the big data evaluation method presents a handover algorithm. The main concern is achieving better user service quality along with load balancing occurrence. Authors in [14] use the fuzzy logic method to propose a vertical handover algorithm to achieve a better quality of service. Handover decisions are taken based on the current applications’ receiving data rates lacking other contextual information. Fuzzy control logic is applied in [15] to achieve an intelligent self-optimizing handover algorithm for fourth generation (4G)/5G HetNets. Although the proposed scheme reduced unnecessary handovers and handover latency, the unbalanced load problem is not a concern. In [16], the authors’ goal is to solve the network selection problem based on finding the accurate values of considering attributes by applying the hesitant fuzzy theory. Weights of the attributes are calculated using the fuzzy AHP (FAHP). Finally, networks’ ranking is performed using the hesitant fuzzy TOPSIS method. Although the users’ preferences for various application types are considered, the problem of achieving load balance is not a concern. The authors in [17] use the Grey Relational Analysis (GRA) method combined with both AHP and entropy weighting methods to rank the available candidates. Although the proposed work succeeded in reducing the number of handovers, multi-user or multi-application scenarios are not considered. In [18], a novel handover algorithm based on integrating different MADM methods is proposed. The network selection process combines scores obtained by adopting various methods and choosing the network with the highest score. Although the suggested method achieves good results regarding HetNet performance, changes in attributes’ values are not considered in the weighting process, yielding to not being connected to the best network all the time. In [19], a new model for selecting the best network is presented based on a technique known as Improved-MEREC-TOPSIS. The proposed model concentrates on reducing unnecessary handovers. The authors in [20] focus on reducing unnecessary handovers by combining optimization techniques with the fuzzy system to propose a network selection algorithm. Various attributes form a cost function, while the corresponding weights are optimized by particle swarm optimization (PSO). The preferred network for each user of a specific application is calculated using a fuzzy system. A genetic algorithm is used in [21] to optimize the network parameters’ values for decreasing the number of handovers that occur. Authors in [22] use a methodology combining an artificial intelligence approach, fuzzy logic, and artificial bee colony to determine whenever a handover is needed and the best network to be accessed. Despite using various factors involving network parameters and user preferences to make a correct decision, no concern is given for achieving a balanced load. In [23], a handover algorithm is proposed based on a simulated annealing technique to achieve better service quality by finding optimum attribute weights. To reach the best network, authors in [24] use a prediction technique with the PSO algorithm and propose an algorithm based on the multiobject-PSO method, optimizing attributes weights, and finally, the network with the lowest cost function is selected. Authors in [25] focus on the handover process as a real-time problem, so they propose an artificial bee colony (ABC) algorithm consisting of various objective functions. The presented algorithm finds the best network in less time than similar algorithms. An intelligent handover algorithm based on two heuristic algorithms presented as the ABC-PSO algorithm is introduced in [26]. The authors’ goal is the assignment to the suited network while performing as few handover numbers as possible. To reduce the chance of handover failure while keeping the number of unnecessary handovers at a low level, a handover algorithm based on the ant colony optimization technique is presented in [27]. The authors in [28] modify the handover algorithm based on the weed optimization (WO) technique, which was introduced in [29], to enhance the network selection process by saving the mobile battery and reducing unnecessary handovers while achieving a fair load distribution.

Due to the users’ mobility and the changes in the network situation regarding attribute values and loading status, an automated handover algorithm that can investigate all the matter factors and adjust itself during the running process becomes necessary. In this paper, an enhanced adaptive optimized vertical handover algorithm is proposed. We enhance the earlier work introduced in [18] on multiple levels. We extend the heterogeneous network environment by including the 5G network along with considering additional necessary network attributes. Combining various strategies in searching for the best network is performed as successive steps to exclude any unsuited network earlier and avoid additional computations. Weighting considered attributes is performed using both AHP and entropy methods to represent users’ preferences along with different attributes’ objectivity aiming to guarantee scoring candidate networks correctly. To ensure users’ desires are achieved, two new control parameters are introduced to enhance the handover process performance concerning HetNet’s point of view, aiming to reduce unnecessary handovers and keep involved networks uncrowded. The handover problem is solved as an optimization framework. The proposed algorithm is assumed to be run in a centralized coordination entity in the HetNet, which controls all associated networks and users. The proposed handover algorithm’s adaptation capability makes it suitable for being applied in cognitive radio heterogeneous networks and so emphasizes the presented work strength in achieving better Quality of Service (QoS) for users and enhances the network performance in many sights other than similar existing work.

Contributions of this paper can be summarized as follows:

1. A novel enhanced network selection method is introduced based on gathering user preferences and network characteristics to reach the best network accurately. The selection process involves many network selection strategies combined with each other for specifying potential candidate networks with utilizing all the benefits.
2. Two new handover control parameters are introduced for reducing unnecessary handovers and solving unbalanced load problems. The two parameters are named the score priority (SP) parameter, which represents the permissible decreasing amount in the connected network score with no need to perform handover, and the load factor (LF) parameter, which represents the maximum amount of bandwidth to be occupied for each network and so prevents overloaded network problem.
3. An adaptive optimized vertical handover algorithm involving optimal handover control parameters values selection approach using particle swarm optimization and gravitational search algorithm (PSOGSA) is proposed.
4. The simulation results validate the efficient performance of the proposed algorithm by decreasing unnecessary handovers by more than 40% and achieving much better load distribution by around 20% to 40%.

This paper is organized as follows: The System model and problem statement section describes the proposed system model and the problem statement of the proposed work. The Methods section illustrates the involved methods for the network selection process, introduces the optimization approach to solve previously discussed problems, and describes the optimization algorithms involved in the proposed work. The simulation results are illustrated with a discussion in the Results and discussions section. Finally, the main conclusions and future work are presented in the Conclusion and future work section.

## System model and problem statement

### Network model

**Fig 1:**
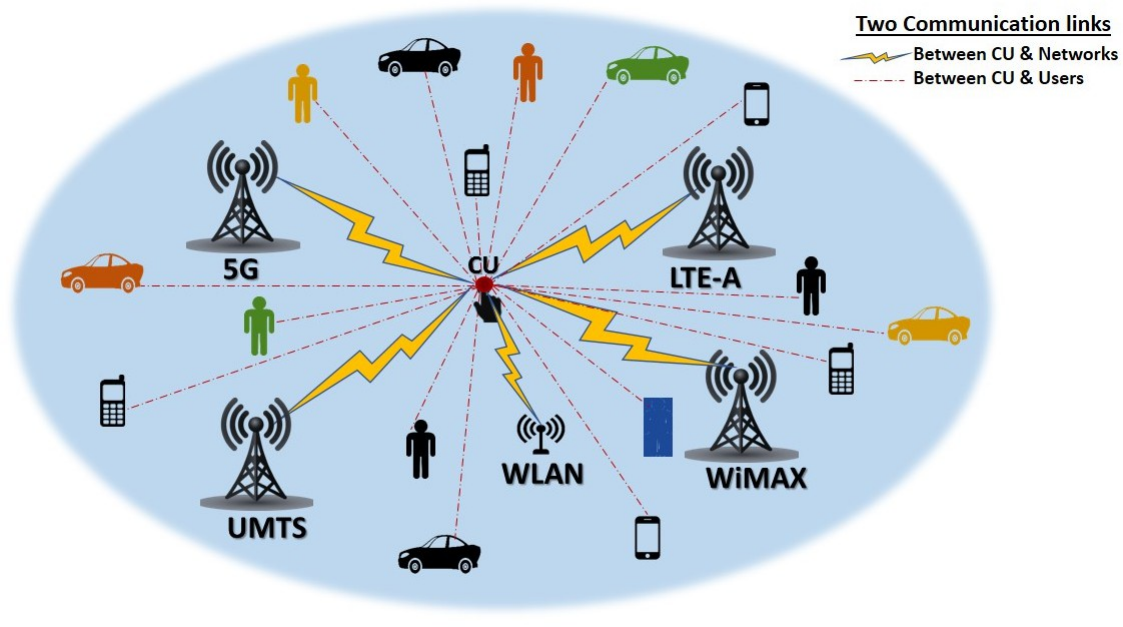
System model.

We consider a heterogeneous network constructed of five overlapping main networks, 5G, LTE-A, WiMAX, UMTS, and WLAN, with a centralized control unit (CU) where mobile users of various applications exist, as shown in Fig 1. It is assumed that the CU can communicate with all involved networks and users to gather all essential information required to perform the handover process [30]. The goal is to assign a suitable network for each user with a single application type of either voice, video, or data while moving across the considered HetNet. The considered attributes are available bandwidth, maximum delay time, jitter time, loss, and price.

To find the best-suited network according to each user preference and all available networks’ characteristics, we propose Algorithm 1 to represent the initial sequence of the proposed network selection process. In Algorithm 1, the received signal power for surrounding networks is calculated corresponding to the requested handover user to configure the candidate networks with an RSS value above the specified threshold. The received power value, which is affected by the path loss, is calculated in the presented model using the Okumara-Hata model [31]. As a higher handover number yields in increasing signal overhead, to reduce unnecessary handovers, the proposed algorithm excludes WLAN from the serving opportunity of users moving with high speed due to the expected short serving time. Scoring candidate networks is performed by adopting the MADM strategy. The handover decision is taken to the network of the highest score with an RSS value above the specified threshold.

### Problem formulation

Although connecting each user to the highest score network will guarantee achieving the desired satisfaction according to users’ demands, this may lead to degrading the HetNet performance as follows:

1. Fluctuations in the attributes’ values due to the user’s mobility and any changes in the surrounding environment during successive times will affect the networks’ ranking and increase the handovers’ number, including unnecessary handovers referred to as ping pong handovers.
2. Also, suppose one or two networks are the preferable ones for most users due to their high scores. In that case, this will cause unbalanced load distribution, and the congested networks will suffer from performance degradation, which may cause connection failure.
3. An important factor that needs to be considered is the amount of power consumption in the HetNet. Increasing power consumption will lead to a waste of resources in addition to increasing the required cost.

**Algorithm 1: Main Network Selection algorithm**

Initialization of the HetNet. Create mobile stations with multi-interface. Select randomly mobile stations’ position, speed, and traffic type.

**1 begin**

**2**  **for** t = 1: No. of time samples

**3**   **for** N = 1: No. of users

**4**    Calculate RSS of all available networks, configure networks of RSS ≥ RSS_th_

**5**    **if** only one network is detected

**6**     **then** connect UE to this network

**7**    **else if** more than one network detected

**8**     **if** UE speed is high

**9**      **then** remove WLAN if found from the candidate networks

**10**      **else** keep WLAN

**11**     **end**

**12**    **then**

**13**     Configure candidate networks’ attributes

**14**     Calculate networks’ scores based on their weighted attributes

**15**     Sort candidate networks in descending order according to their scores

**16**     Establish a connection to the first network in the ranking list

**17**    **end**

**18**   **end**

**19**  **end**

**20 end**

Next, we will express the above-stated problems as parameters to be measured and used in the performance evaluation of the proposed algorithm. The notations used in this paper are summarized in Table 1.

**Table 1:** List of notations.

Symbol	Definition
N	The number of candidate networks.
n	The number of attributes.
*r*_*im*_	The normalized value of attribute i for network m.
*Y*_*im*_	The original value of attribute i for network m.
*d*_*im*_	The rate of network m for attribute i.
*e*_*i*_	The entropy of attribute i.
*w*_i_	Weight of attribute i.
*U*_*im*_	The utility value of attribute i.
*D* ^-^_*im*_	The distance from network m to the worst network regarding attribute i.
*D* ^+^_*im*_	The distance from network m to the best network regarding attribute i.
*X* ^+^_*im*_	The best value of attribute i.
*X* ^-^_*im*_	The worst value of attribute i.
*P*_*C*_	The power consumption of HetNet.
*r*	The Network radius.
*P*_*t*_	The network transmitted power.
*G*_*t*_	The network transmitter gain.
*G*_*r*_	The network receiver gain.
*f*	The network operating frequency.
*h*_*m*_	The mobile station antenna height.
*h*_*b*_	The base station antenna height.
*RSS*_*th*_	The received signal strength threshold.
*P*_*trans*_	The power consumption of the transceiver.
*P*_*amp*_	The power consumption of the power amplifier.
*P*_*proc*_	The power consumption of digital signal processing.
*P*_*rect*_	The power consumption of the rectifier.
*P*_*link*_	The power consumption of the microwave link.
*P*_*air*_	The power consumption of the air conditioning.

#### Evaluation of HetNet performance

Four parameters; handover (HO) number, ping-pong (PP) number, the coefficient of variation (CoV), and the power consumption of HetNet (P_C_) are considered while evaluating the proposed VHO algorithm’s performance and can be defined as follows:

- HO number; represents the times of changing the user’s connected network for any two consecutive time samples.
- PP number; represents times when a user is handed over from one network to another and returned to the original one in the next time sample.
- CoV, calculated by (1); can be considered as a measurement of the uniformity of load distribution among all the HetNet participating networks.
$$CoV=\frac{load\prime s\;Standard\;Deviation}{Mean\;load}$$ (1)
- Power Consumption of HetNet (P_C_); in the proposed work will be represented by (2); as around 60% of power dissipated in the HetNet will result corresponding to base stations power transmitted [32].
$$P_{C}=L\left(P_{trans}+P_{amp}+P_{proc}\right)+P_{rect}+P_{link}+P_{air}$$ (2)
  where L represents the ratio of the network occupying bandwidth to the total bandwidth, equals1 in case of full load situation, while P_trans_, P_amp_, P_proc_, P_rect_, P_link_, and P_air_ are the power consumption (in Watt) of the transceiver, the power amplifier, the digital signal processing, the rectifier, the microwave link, and the air conditioning, respectively [33].

According to Algorithm 1, any changes in network scores during decision times will specify which network to be connected to, i.e., if the score of the connected network is decreased even with a small value, the user will be connected to another network leading to increase both HO number and PP number. Also, both load congestion and high-power consumption problems appear, represented by higher values of CoV and Pc, in the case of a persistence ranking list affecting the taken handover decision.

#### Proposed solution

As a try to overcome higher HO and PP numbers, we introduce the score priority (SP) parameter to give the connected network priority when selecting the best network according to scores in case the decrease in its score value is small in a way that will not affect the user satisfaction about the running service. As shown in (3); a handover is executed when the difference between the score of the first-ranked network and the score of the connected network is greater than SP; otherwise, the existing network connection is maintained, which can reduce unnecessary handovers.


$$Score_{first\;ranked\;network}-Score_{connected\;network}>SP$$ (3)

Regarding the unbalanced load problem, we introduce another parameter called load factor (LF), representing the maximum bandwidth occupied. Looking closely at (1), we can see that whenever load distribution over candidate networks changes, the load’s standard deviation value changes while the mean load value remains constant; as for N networks.


$$load^{\prime }s\;Standard\;\;Deviation=\sqrt{\sum_{m=1}^{N}{\left(Occupied\;load_{m}-Mean\;load\right)}^{2}}$$ (4)

So, controlling the occupied amount of load will prevent preferable networks from being highly congested by offloading them to the next preferred networks and achieving a much more uniform load distribution yielding to smaller CoV value as the difference between each network load and the mean load is reduced. Also, from (2); the occupied amount of load for each network, represented by L, affects the total HetNet power consumption, as multiplying by a smaller value for L will decrease the power consumption. The modified handover algorithm is represented by Algorithm 2.

The process of offloading a congested network, controlled by the LF value, conflicts with keeping unnecessary handovers as small as possible, which is controlled by the SP value. To enhance the presented work and reach a handover algorithm able to adapt itself and make precise changes whenever needed to increase the efficiency of the HetNet performance while maintaining acceptable user satisfaction, an optimization phase is introduced to the algorithm procedure to obtain the optimum values of both LF and SP parameters. To represent all cases of occupation, searching for the optimum value of the LF parameter is performed in a range of 0 to 1. Using the normalized score in ranking candidate networks, the score’s fluctuation in the range of 0.7 to 1 could be represented as a satisfied score. So, searching for the optimum value of the SP parameter is performed in the range of 0.01 to 0.3 to represent an acceptable decrement in the connected network’s score.

All the evaluation parameters are combined to represent one objective function defined as

MinF

where

$$F=\frac{P.P.}{HO}+CoV+P_{C}$$

*Subject to*:

$$\begin{array}{l} 0<LF\leq 1\\ 0<SP\leq 0.3 \end{array}$$ (5)

**Algorithm 2: Proposed modified handover algorithm**

**Input**: Initialize the HetNet system model

**Output**: Handover number, PP number, CoV, and HetNet power consumption

**1 begin**

**2**  **for** t = 1: No. of time samples

**3**   **for** N = 1: No. of users

**4**  Follow Algorithm 1 Procedure from line 4 to line 12

**5**    Configure candidate networks

**6**    **then**

**7**     Check each candidate network’s load

**8**     Remove network with load index = LF

**9**     Configure candidate networks attributes

**10**     Calculate combined weights of different criteria

**11**     Calculate networks scores

**12**     Configure the connected network of the previous time sample **then** add SP value to its score

**13**     Sort candidate networks in descending order

**14**     Establish a connection to the first network in the ranking list

**15**     **if** the currently connected network is the same previous one

**16**      **then** keep no. of handovers (HO) the same

**17**      **else** increment HO by one

**18**     **end**

**19**     Check the connected network before the past one

**20**     **if** it is the same current network

**21**      **then** increment no. of ping pong (PP) by one

**22**      **else** keep PP no. the same

**23**     **end**

**24**    **end**

**25**   **end**

**26**   Compute the load index of each network

**27**   **end**

**28**   **return** HO, PP number, CoV, and HetNet power consumption

**29**  **end**

## Methods

Using any MADM method to determine the network to be assigned by a specific user requires ranking the candidate networks according to their weighted attributes’ scores. The weight value given to an attribute depends on its importance for the user’s preference. For benefit attributes, weight is preferred to be the highest as possible while it should be kept in small value for non-benefit ones. In the upcoming sub-sections, various methods used for attribute weighting and scoring adopted by the proposed work, are described. The details of how these methods are implemented and their rationale behind their selection will be elaborated on.

### Calculate the weights of network attributes

Over the past years, various weighting methods have been proposed for solving multi-criteria decision problems, such as AHP and entropy methods, which are explained next.

#### 1) Weighting using the AHP method

**Fig 2:**
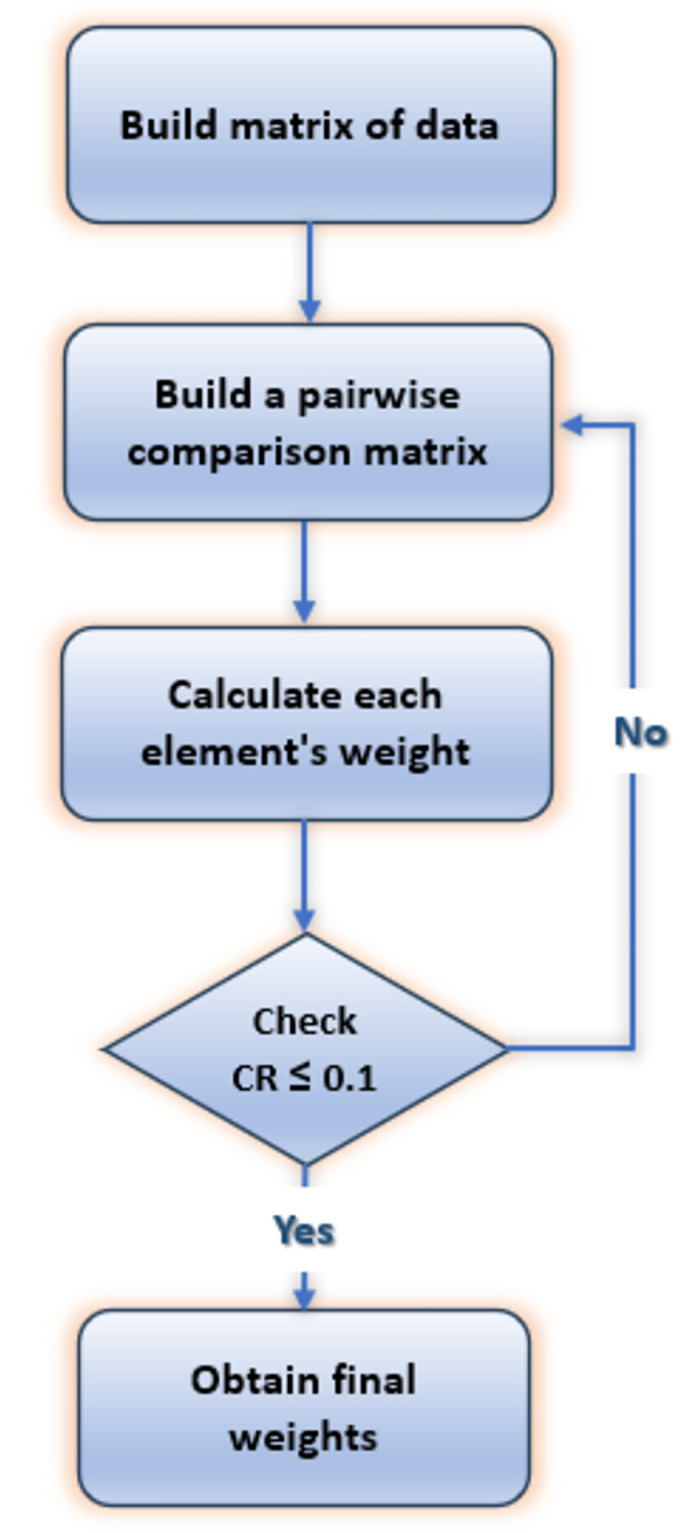
Flowchart of weighting using the AHP method.

Due to the strength of the AHP method, developed by Saaty [34], in allowing some small inconsistencies in making decisions, it has been widely applied to MADM problems in various fields. The flow chart of the weights’ calculation steps is shown in Fig 2 and can be summarized as:

1. Making pairwise comparisons of attributes due to their relative importance represented in matrix form and scaled as in Table 2, according to differences in users’ preferences for considered applications.
2. For n attributes, the weight of an attribute corresponding to a specific row equals the normalized value of the nth root of the product of its row elements.
3. As the last step, verification of the comparisons’ consistency is performed by checking the consistency ratio (CR) value if it is below 10%; the obtained weights are considered acceptable.
  The consistency ratio (CR) is calculated as in [10]
$$CR=\frac{CI}{RI}$$ (6)
  where CI is the consistency index, and RI is the random index of consistency, and its value is assigned according to the number of attributes (n) as in Table 3. According to Saaty; for less than three attributes, the RI value is zero meaning the comparisons must be consistent.

**Table 2:** Saaty’s scale of importance.

Scale	Importance value
9	Extreme
7	Very Strong
5	Strong
3	Moderate
1	Equal
8,6,4,2	Intermediate

**Table 3:** RI value according to the number of attributes.

**n**	2	3	4	5	6	7	8	9
**RI**	0	0.52	0.9	1.11	1.24	1.35	1.41	1.45

CI can be calculated by (7) [10]

$$CI=\frac{\lambda_{max}-n}{n-1}$$ (7)

where λ_max_ is the maximum Eigenvalue of the built pairwise comparison matrix [35].

#### 2) Weighting using the entropy method

**Fig 3:**
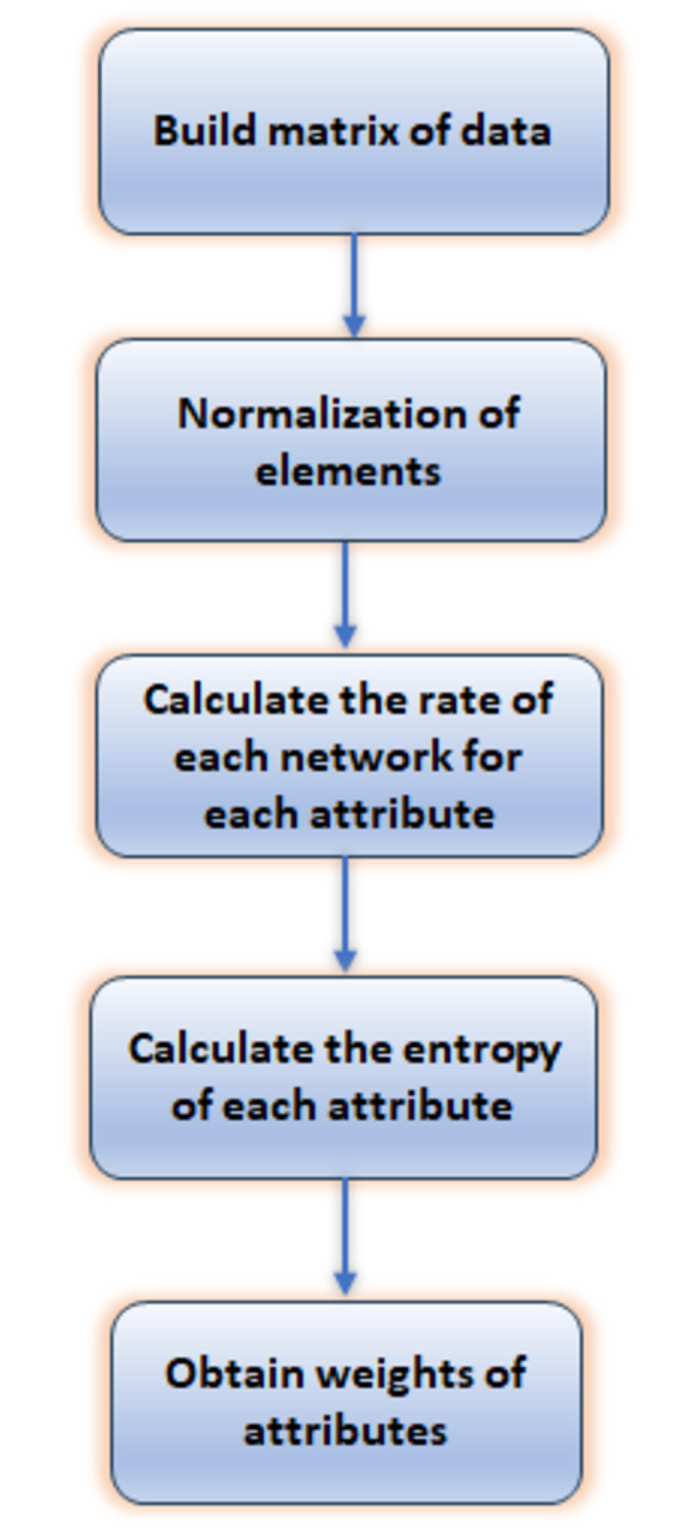
Flowchart of weighting using the entropy method.

The entropy method for weighting is based on information entropy, a measurement of the difference between compared systems, proposed by Shannon. Because of its benefits of easiness and accuracy, it is commonly used [36]. By using the entropy method, each attribute’s weight is obtained according to the gathered data without any consideration of the user’s preference or experience. The flow chart of the weights’ calculation steps is shown in Fig 3 and can be summarized as:

1. As the attributes considered in the decision process differ in their measurements’ units, normalizing the decision matrix elements is performed using (8) and (9) for benefit criteria and non-benefit criteria, respectively [36].
$$r_{im}=\frac{Y_{im}-\text{min}\;Y_{im}}{\text{max}\;Y_{im}-minY_{im}}$$ (8)
$$r_{im}=\frac{maxY_{im}-Y_{im}}{\text{max}\;Y_{im}-minY_{im}}$$ (9)
  where *r*_*im*_ is the normalized value of the i^th^ attribute for candidate network *m*, and *Y*_*im*_ is the original attribute value.
2. The second step is to compute the rate of each candidate network when considering each attribute individually, as shown in (10) [17]
$$d_{im}=\frac{r_{im}}{\sum_{m=1}^{N}r_{im}}$$ (10)
  where *d*_*im*_ is the rate of the m^th^ network considering the i^th^ attribute.
3. Next, the entropy of the i^th^ attribute is calculated as in (11) [17].
$$e_{i}=-h*\sum_{m=1}^{N}d_{im}*ln\;d_{im}$$ (11)
  where the value of coefficient *h* depends on the number of candidate networks (N) and equals 1/lnN [17].
4. Finally, the weight of each attribute is calculated by (12) [17].
$$w_{i}=\frac{1-e_{i}}{\sum_{i=1}^{n}1-e_{i}}$$ (12)
  In the next subsection, the adopted scoring strategies are discussed in detail.

### Scoring candidate networks

**Fig 4:**
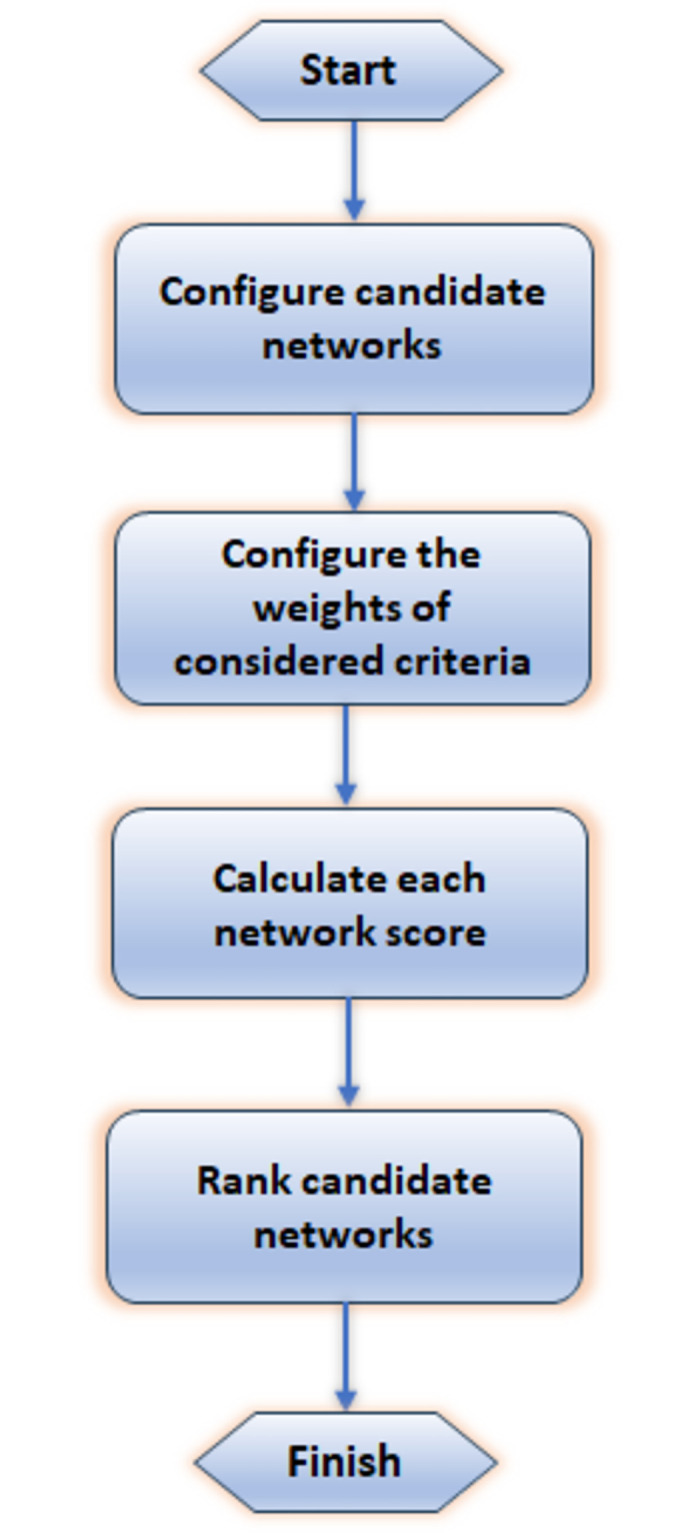
MADM methods’ general flowchart.

Different scoring strategies are investigated. Each strategy is applied to score the available networks and then specify the best network with the highest score to be connected by the corresponding users at each decision time. A general flow chart of MADM methods is shown in Fig 4. The steps of each adopted strategy are explained next.

To satisfy the served users by a reasonable degree, utility theory represents the values of the attributes. Describing a criterion’s effect on a single application type by a suited utility function is presented in many studies [37, 38].

All concerning attributes’ utility values are determined and then weighted by their corresponding weights. Scoring networks using the utility functions determined by [37]

$$Score_{utility}=\;\sum_{i=1}^{n}w_{i}U_{im}$$ (13)

where *n*, *w*_*i*_, and *U*_*im*_ are the number of attributes, the weight of the *i*^*th*^ attribute, and the utility value of the *i*^*th*^ attribute, respectively for the m^th^ network.

Different utility functions are adopted for each attribute according to the considered applications, as shown in Table 4, where *Y*_*im*_ is the attribute value. Linear decreasing functions are used for all considered applications to represent the effect that when either the packet loss or the service price increases, the satisfaction degree decreases. Sigmoid functions are used for representing delay utility functions in the case of all applications and for representing the bandwidth effect in the case of voice and video applications, while an exponential function is used for data applications. Regarding the jitter effect, voice and video applications are more sensitive than data applications, so logarithm functions are used for them while using a linear decreasing function for data applications.

**Table 4:** Utility functions for attributes for different applications.

	Voice	Video	Data
**Bandwidth**	$U_{im}=\frac{({Y_{im})}^{5}}{1+({Y_{im})}^{5}}$	$U_{im}=\frac{({\frac{Y_{im}}{2.5})}^{5}}{1+({\frac{Y_{im}}{2.5})}^{5}}$	$U_{im}=\frac{e^{0.5Y_{im}}-1}{e^{0.5Y_{im}}}$
**Delay**	$U_{im}=1-\frac{({\frac{Y_{im}}{50})}^{5}}{1+({\frac{Y_{im}}{50})}^{5}}$	$U_{im}=1-\frac{({\frac{Y_{im}}{100})}^{5}}{1+({\frac{Y_{im}}{100})}^{5}}$	$U_{im}=1-\frac{({\frac{Y_{im}}{150})}^{2.5}}{1+({\frac{Y_{im}}{150})}^{2.5}}$
**Jitter**	$U_{im}=1-(-2.76+0.75*\text{ln}\left(Y_{im}+35\right))$	$U_{im}=1-(-1.35+0.5*\text{ln}\left(Y_{im}+15\right))$	$U_{im}=1-\frac{Y_{im}}{100}$
**Loss**	$U_{im}=1-\frac{Y_{im}}{25}$	$U_{im}=1-\frac{Y_{im}}{25}$	$U_{im}=1-\frac{Y_{im}}{25}$
**Price**	$U_{im}=1-\frac{Y_{im}}{80}$	$U_{im}=1-\frac{Y_{im}}{90}$	$U_{im}=1-\frac{Y_{im}}{100}$

SAW is a famous MADM technique that requires adjustment of the attributes’ values, weighting them, and finally summing the weighted adjusted values to find the corresponding network score as in (14) [39].

$$Score_{SAW}=\;\sum_{i=1}^{n}w_{i}r_{im}$$ (14)

where r_*im*_ is an adjustable value, through the normalization process, of the *i*^*th*^ attribute.

WPM is similar to SAW in normalizing the attributes’ values but scoring by WPM, shown in (15), is different as it represents the multiplication of the normalized attributes raised to a power equal to their relative weights individually [40].


$$Score_{WPM}=\;\prod_{i=1}^{n}r_{im}{}^{w_{i}}$$ (15)

TOPSIS is the last method that participates in our model. The closeness to the best solution must be determined to score the alternative networks as in (16) [41].

$$Score_{TOPSIS}=\frac{D_{im}^{-}}{D_{im}^{-}+D_{im}^{+}}$$ (16)

where *D*^-^_*im*_ and *D*^+^_*im*_ are the distances from the alternative network to the worst and the best ones and are calculated using (17) and (18) [41].

$$D_{im}^{+}=\sqrt{\sum_{i=1}^{n}{\left(X_{im}^{+}-X_{im}\right)}^{2}}$$ (17)

$$D_{im}^{-}=\sqrt{\sum_{i=1}^{n}{\left(X_{im}^{-}-X_{im}\right)}^{2}}$$ (18)

where *X*_*im*_ = *w*_*i*_ *r*_*im*_, *Y*_*im*_ is the attribute value, and *r*_*im*_ is the normalized value, which is calculated by (19) [41].

$$r_{im}=\frac{Y_{im}}{\sqrt{\sum_{m=1}^{N}Y_{im}^{2}}}$$ (19)

while $X_{im}^{+}$, and $X_{im}^{-}$, i = 1, 2, 3, …, n, are the attributes of the best and the worst network. Their values could be the minimum or the maximum value among the attribute’s values of all the networks depending on whether it is a benefit or a non-benefit one [42].

### Proposed optimized handover algorithm (PSOGSAHO)

Heuristic optimization algorithms such as particle swarm optimization (PSO) algorithm and gravitational search algorithm (GSA) have been recently used in the field of heterogeneous networks to reach the best network that optimally fulfills the user-requested QoS with guaranteeing efficient performance regarding the demanded objectives [43, 44]. The goal is to find the optimal outcome among all potential inputs out of the search space by applying an effective search procedure. Combining optimization algorithms in one model is considered a good solution to compromise the ability of both exploration and exploitation to rate up the algorithm performance [45].

We have proposed the PSOGSA as a hybrid algorithm from combining PSO and GSA algorithms in parallel run by Mirjalili and Hashim in [45], presenting it as one approach to solve the optimization problem and obtain the optimum solution, representing the optimum LF and SP values.

#### PSOGSA

**Fig 5:**
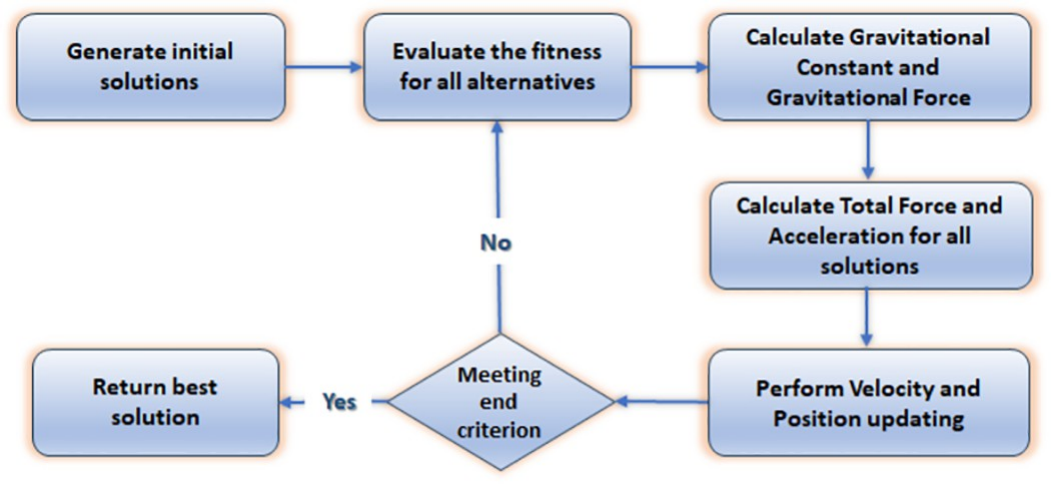
Flow chart of the PSOGSA scenario.

In a PSO system, particles move in the search space while adjusting the position of each particle depending on the experiences of itself and an adjacent particle, called pbest, and gbest, respectively. The principle of GSA considers the proportional relation between the masses of the possible solutions and the values of their fitness function. The resulting gravity forces cause attraction directly proportional to the related mass size [43, 44]. The PSOGSA combines both algorithms’ strengths to find the optimal solution. A flow chart of the PSOGSA procedure is shown in Fig 5.

The PSOGSA works on the following steps:

- First, all alternative solutions are randomly distributed in the search space.
- The best-found solution is saved for all running iterations to be accessed whenever needed. Along the whole algorithm, the position of each alternative is updated sequentially by calculating the velocity and acceleration as follows [44].
$$X_{new}=X_{past}+V_{new}$$ (20)
  where *X*_*new*_ and *V*_*new*_ are the updated position and velocity.
$$V_{new}=wV_{past}+c_{1}\times q\times acc_{past}+c_{2}\times q\times \left(g_{best}-X_{past}\right)$$ (21)
  where *w* represents the inertia weight. *c*_*1*_ and *c*_*2*_ are the acceleration coefficients. *q* is a random number in the interval [0, 1], *acc*_*past*_ is the past iteration acceleration, and *g*_*best*_ is the optimum solution found by all candidates.
  The low of motion is used to calculate the acceleration of any candidate at any iteration as
acc=FM (22)
  where *M* is the candidate’s inertial mass while *F* represents the total affected force on a single candidate by the other ones. For N total candidates, the force affected on candidate j is determined by
$$F=\;\sum_{i=1,j\neq i}^{N}q_{i}F_{ji}$$ (23)
  where *Fji* is the gravitational force on candidate *j* from candidate *i* while *q*_*i*_ is a random number, calculated for every candidate, between [0, 1].
  The *Fji* is calculated as (24):
$$F_{ji}=G\frac{M_{j}M_{i}}{R_{ji}+\varepsilon }\left(X_{i}-X_{j}\right)$$ (24)
  where *G* is the gravitational constant, *M*_*j*_ is the gravitational mass of the affected candidate *j*, *M*_*i*_ is the gravitational mass of the acted candidate *i*, *R*_*ji*_ is the Euclidian distance between the two concerning candidates *j* and *i*, ε is a small constant, *X*_*i*_ and *X*_*j*_ are the positions of the acted and affected candidates respectively. The gravitational constant *G* is determined by
G=G0×e−γtmaxt (25)
  where *G*_*0*_ and *ɣ* represent the initial value of the gravitational constant and a constant value while *t* and *max t* are the current iteration number and the iterations’ maximum number, respectively.
- Velocities and positions will continue updating tell reaching the maximum number of iterations and the end limit of a considered criterion is met.

#### PSOGSA approach for the proposed PSOGSAHO

**Fig 6:**
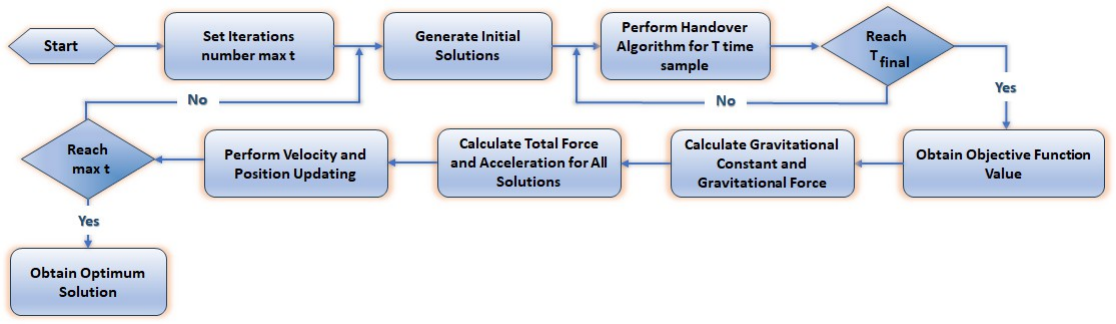
The flow chart of the proposed PSOGSAHO scenario.

The proposed PSOGSAHO algorithm is deployed by including the PSOGSA steps with the proposed modified handover algorithm explained previously. The combined procedure is shown in Fig 6. In the beginning, the number of iterations is determined, generating the PSOGSA initial solutions. While running the handover algorithm, the candidates start to search for the optimal solution according to the proposed objective function. According to the steps of the PSOGSA, all candidates’ total force and acceleration are calculated, followed by updating candidates’ velocity and position. The proposed PSOGSAHO will reach its final step at the end of iterations.

## Results and discussions

### Simulation model

A simulation of the proposed handover scenario is implemented using MATLAB R2019a by a computer with a 3.30 GHz core i7 processor and 16 GB of RAM. Our proposed algorithm is subjected to three stages; one before introducing the earlier mentioned control parameters LF and SP, represented by Algorithm 1, the second with considering them in the handover decision algorithm, represented by Algorithm 2; and the third stage, which is represented by the PSOGSAHO algorithm. The results obtained from the three stages of experiments are discussed in the coming subsection. Comparative charts evaluating the performance of the proposed algorithm are illustrated.

In our work, we have considered a HetNet, as shown in Fig 1, with an area of coverage of 5000 × 5000 m^2^. Tables 5 and 6 show the experiments’ setting parameters, including parameters of the presented HetNet. Regarding users’ mobility, the random waypoint model (RWP) is used, assuming users’ speed is between (6 m/s) for low-speed users and (25 m/s) for high-speed users. To reduce unnecessary handovers due to short serving time, users moving with speeds from 20 to 25 (m/s) are not allowed to be served by WLAN. Considering the cross-layer design of the proposed HetNet model, the attributes’ values are adopted, as shown in Table 7, representing the dynamically changing ranges of the networks’ attributes.

**Table 5:** Simulation parameters.

Parameter	Value
Coverage area	5000 x 5000 m^2^
Mobility model	RWP
Users speed	6–25 m/s
Users number	250–1000
Time samples	300–1000
Running times	50
Optimization phase iterations	50

**Table 6:** HetNet involved networks’ parameters.

Network Parameter	5G	LTE-A	WiMAX	UMTS	WLAN
*r* (km)	2	2.25	1.5	1.5	0.75
*P*_*t*_ (dBm)	40	35	23	15	20
*f* (MHz)	4500	2400	3500	1900	2600
*G*_*t*_ (dB)	10	14	15	10	5
*G*_*r*_ (dB)	3	3	3	3	3
*h*_*b*_ (m)	25	30	30	40	25
*h*_*m*_ (m)	1	1	1	1	1
*RSS*_*th*_ (dBm)	-100	- 90	-100	-110	-95
*P*_*proc*_ (watt)	100	100	100	100	10
*P*_*amp*_ (watt)	50	24.7	20	15	2.4
*P*_*trans*_ (watt)	100	100	100	100	1.8
*P*_*rect*_ (watt)	100	100	100	100	--------
*P*_*air*_ (watt)	225	225	225	60	--------
*P*_*link*_ (watt)	80	80	80	--------	--------

**Table 7:** Candidate networks attribute values.

Attribute	5G	LTE-A	WiMAX	UMTS	WLAN
**Bandwidth (Mbps)**	10–20	3–8	2–6	1–3	4–10
**Max. Delay (ms)**	6–10	20–150	50–250	30–200	90–300
**Jitter (ms)**	0–5	5–15	10–20	20–40	50–80
**loss (%)**	5–15	6–18	9–20	2–10	4–15
**Price (unity)**	10–30	15–40	20–50	10–35	0–30

### Comparative results

**Fig 7:**
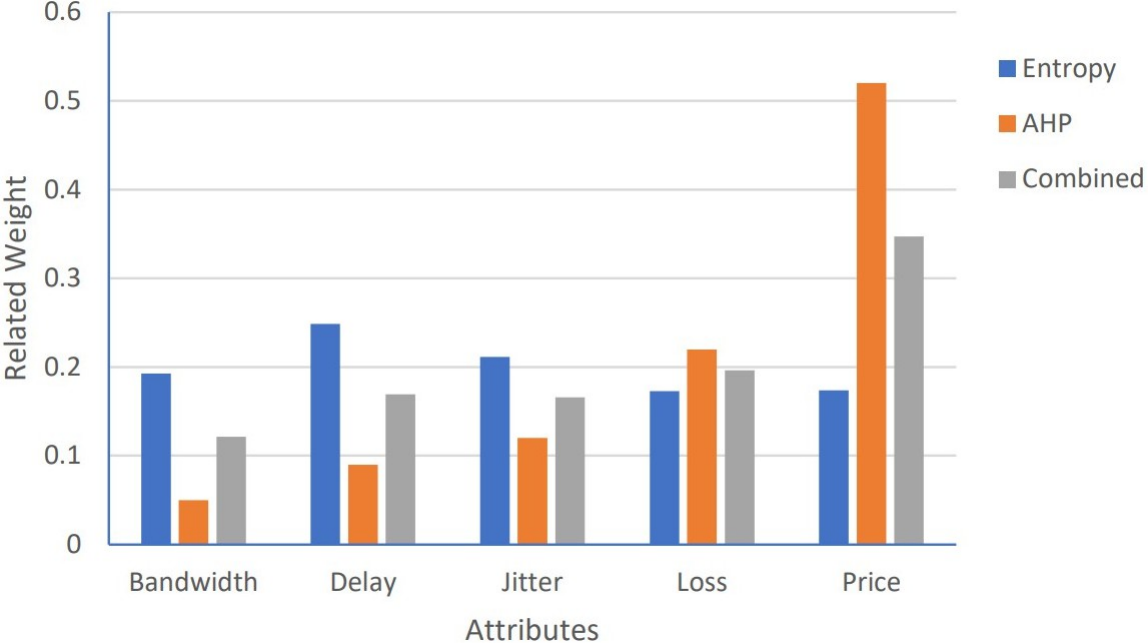
Weights for voice application.

**Fig 8:**
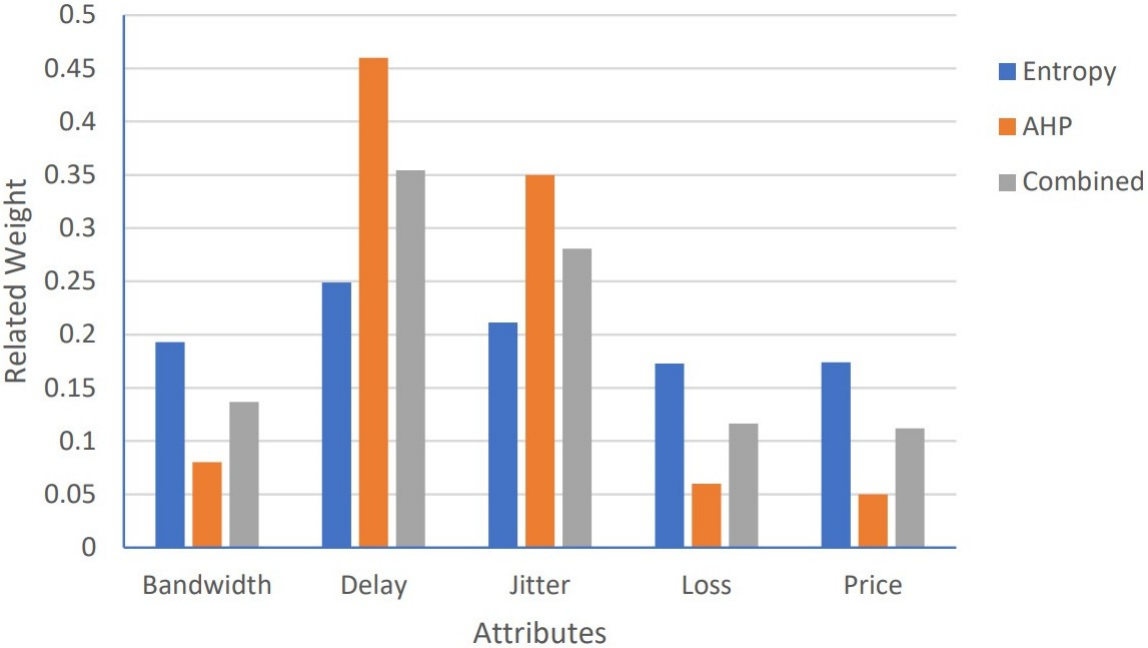
Weights for video application.

**Fig 9:**
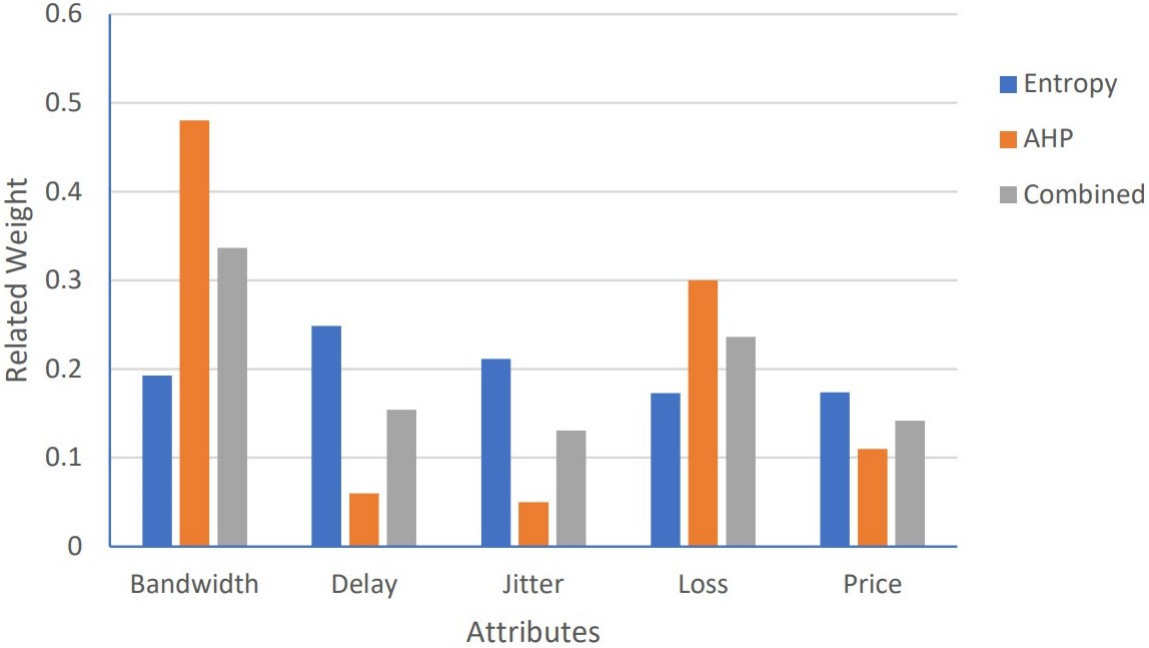
Weights for data application.

To rank the candidate networks accurately, for all proposed algorithms: Algorithm1, Algorithm 2, and PSOGSAHO algorithm, integrated weights as the average of weights obtained from both the AHP and the entropy methods are used; as for the AHP method, the weights depend on the user preferences of the considered attributes while for the entropy method differences between the attribute values are reflected on the calculated weights. Obtained weights for the considered applications according to the candidate networks’ attributes’ values at a certain moment, represented by Table 8, are shown in Figs 7–9.

**Table 8:** Attribute values at a single moment.

Attribute	5G	LTE-A	WiMAX	UMTS	WLAN
**Bandwidth (Mbps)**	15	5	4	2	7
**Max. Delay (ms)**	8	90	150	100	180
**Jitter (ms)**	2	10	15	30	65
**loss (%)**	10	12	15	7	9
**Price (unity)**	20	25	35	22	15

After the weighting attributes step, the candidate networks are ranked. To prove the superiority of the work proposed in this paper, firstly, Algorithm 1 applying TOPSIS, SAW, WPM, and Utility methods individually to rank the candidate networks is compared to classic corresponding MADM algorithms with AHP method used for weighting the attributes, referred to as Algorithm 0. Secondly, the performance of the other successive work stages, Algorithm 2 and PSOGSAHO algorithm is evaluated compared to Algorithm 1 to emphasize each stage achieved enhancement. Comparative charts of the obtained results assuming the proposed system model serves 250 users for 300-time samples are illustrated next.

**Fig 10:**
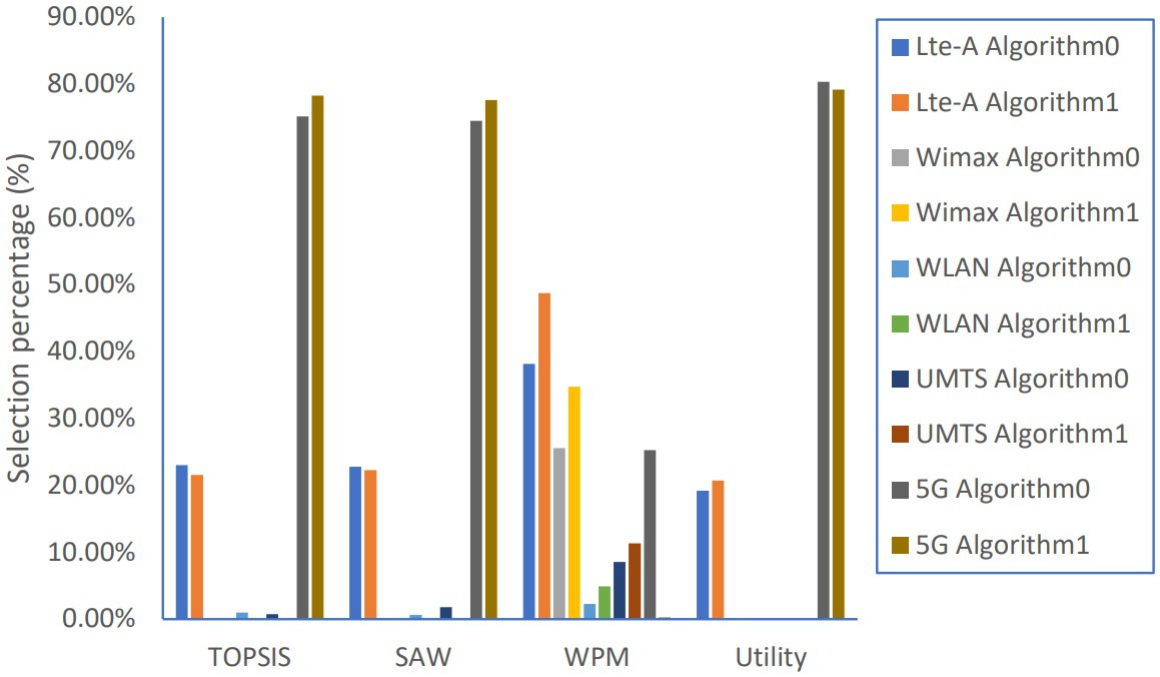
Candidate networks selection percentages.

Average Ranking percentages of all running applications, as the preferable network for all candidate networks corresponding to the applied algorithm, are shown in Fig 10. Because each method scores alternatives with different procedure, the obtained ranking order is different even with the same algorithm; either Algorithm 0 or Algorithm 1 is applied.

**Fig 11:**
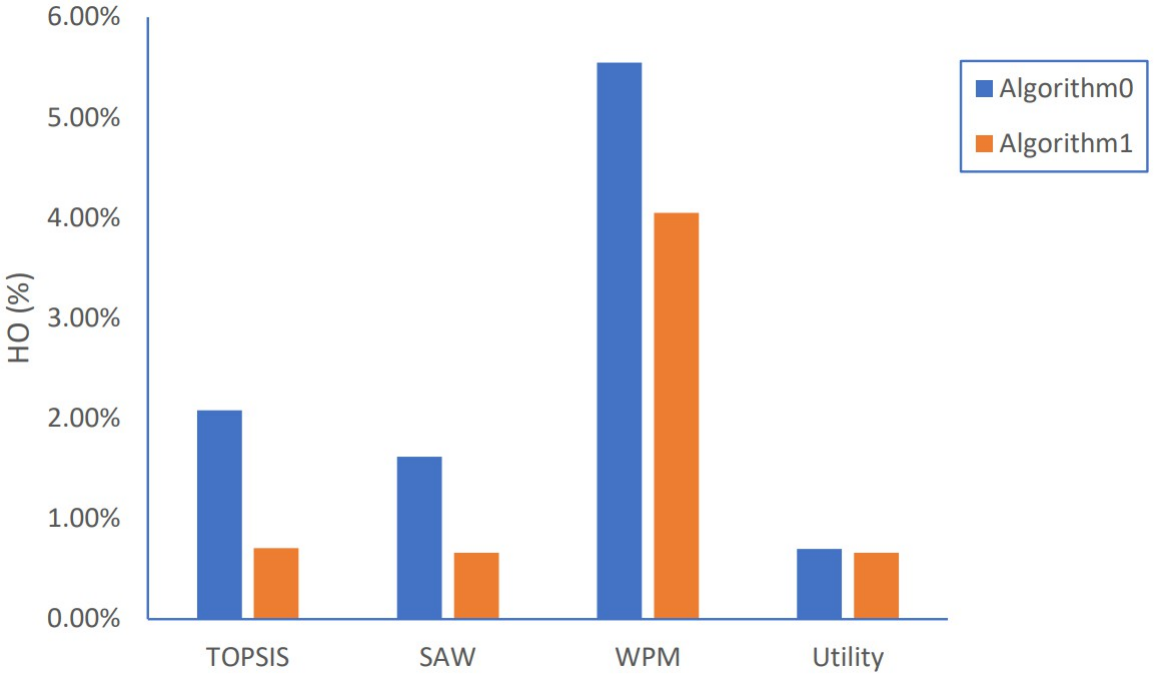
Handover percentage results.

**Fig 12:**
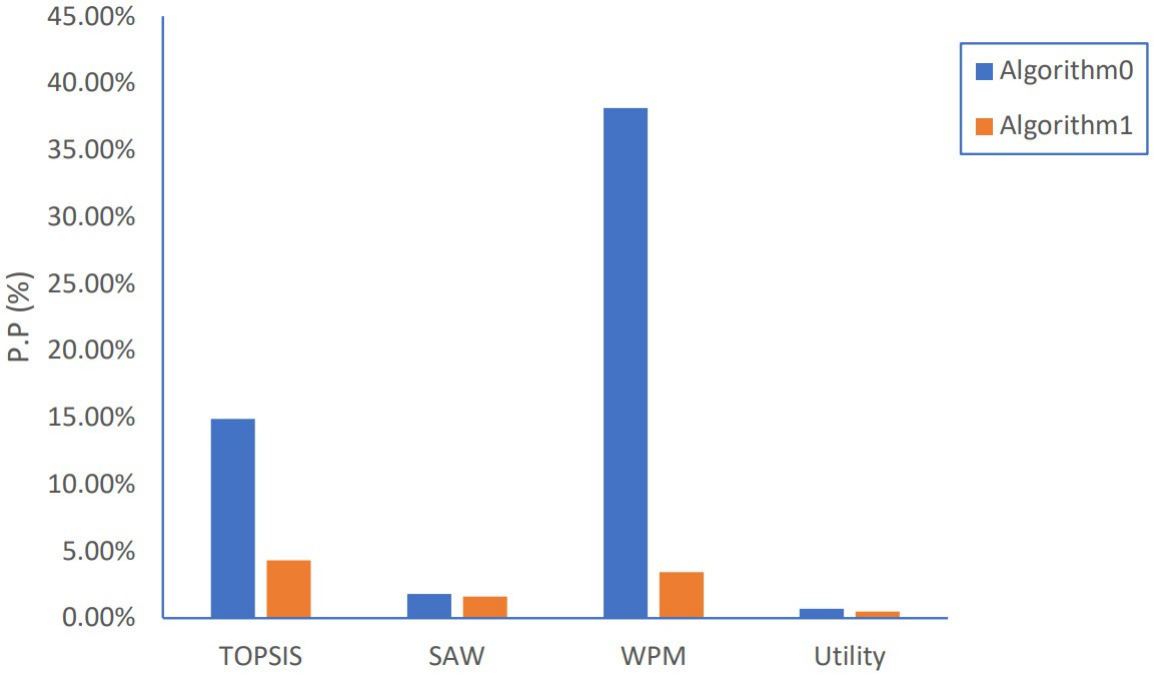
Ping-Pong percentage results.

**Fig 13:**
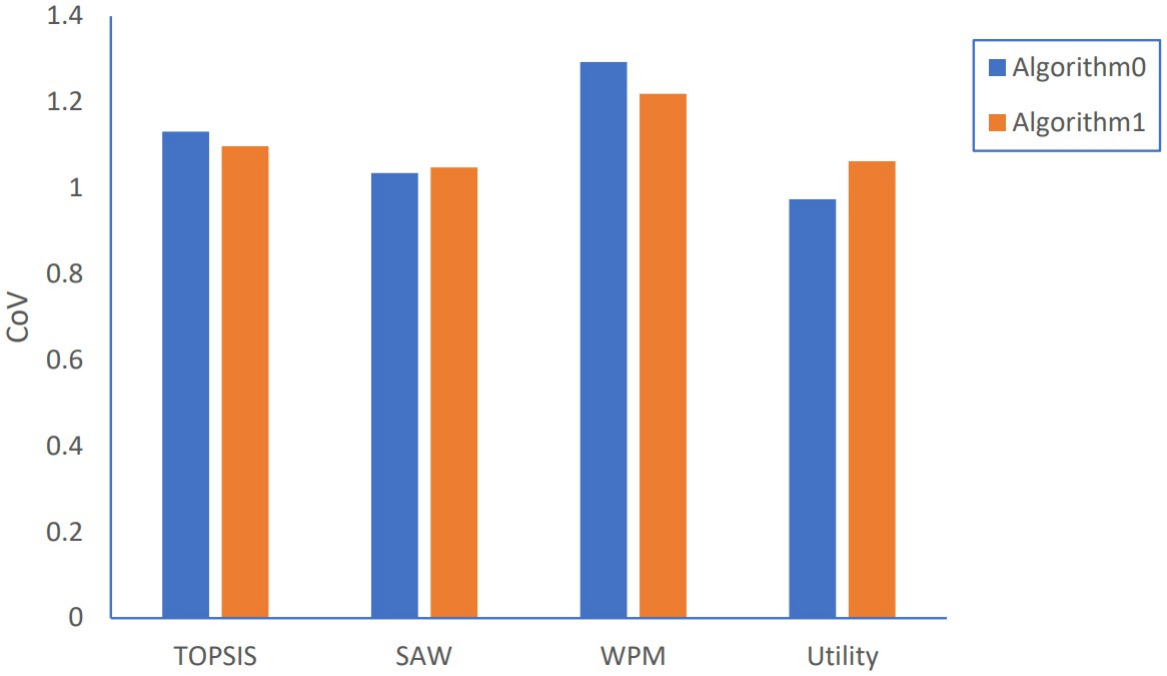
Load distribution results.

**Fig 14:**
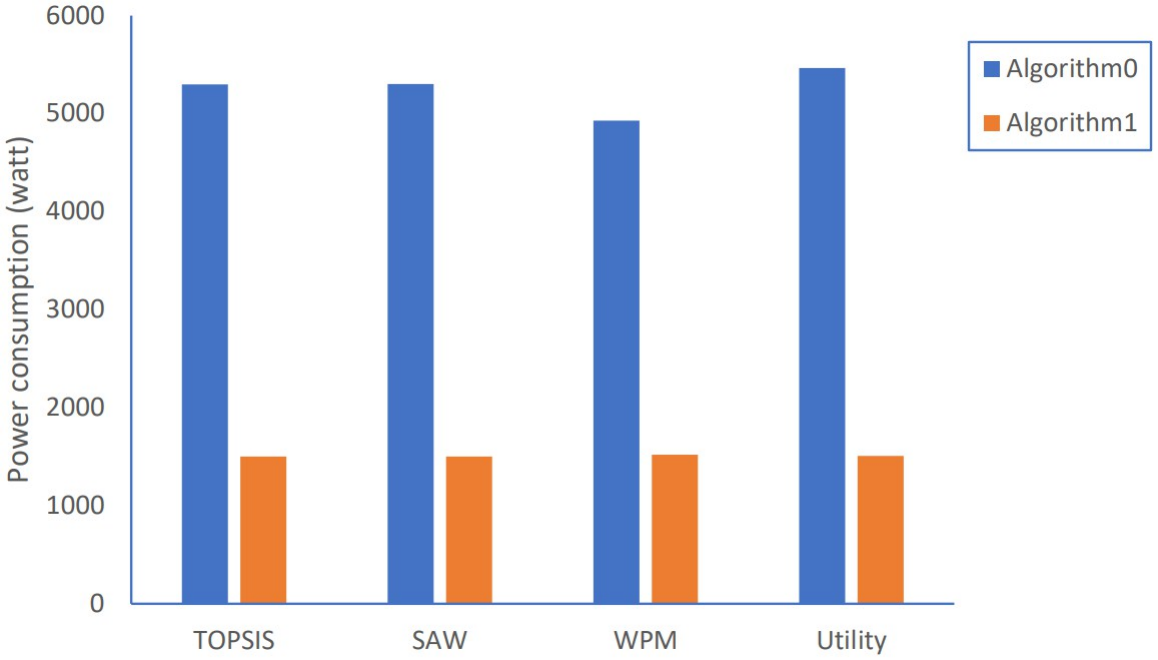
Power consumption results.

For both Algorithm 0 and Algorithm 1, TOPSIS, SAW, and Utility strategies prefer both 5G and LTE-A networks over the other networks ranking the 5G network as the highest score network with almost 80% selection percentage, followed by the LTE-A network with 20%. Using the WPM strategy gives different results when applying Algorithm 0; the most preferred network is the LTE-A network with more than 40%, followed by WiMAX and 5G networks with around 25% for both of them but with applying Algorithm 1; the ranking is changed with considering 5G network the least preferred network while keeping LTE-A and WiMAX networks as the most preferred networks with higher percentages of around 50% and 35% respectively. Each algorithm’s performance reflects These selection percentage changes, as shown in Figs 11–14.

As shown in Fig 11, the proposed Algorithm 1 performs better than Algorithm 0 regarding HO percentage, calculated as the ratio of occurred HO times to the number of handover chances whenever any MADM strategy is applied. TOPSIS strategy obtained the most reduction level with about 65% followed by SAW, WPM, and Utility strategies with reductions of 60%, 27%, and 6%, respectively.

For Ping-Pong percentage, calculated as the ratio of PP number to total HO number, which represents unnecessary handovers, Algorithm 1 has outstanding performance than Algorithm 0, especially for WPM and TOPSIS strategies with reduction level of 90% and 70%, respectively, as shown in Fig 12.

Fig 13 shows the results for load distribution. Algorithm 1 has a slightly better distribution with less CoV value when adopting both TOPSIS and WPM methods while has worse performance with higher CoV value for both Utility and SAW methods.

Regarding the problem of high-power consumption, Algorithm 1 achieves a much smaller average power consumption of almost with about 70% reduction for all adopted strategies, as shown in Fig 14.

**Fig 15:**
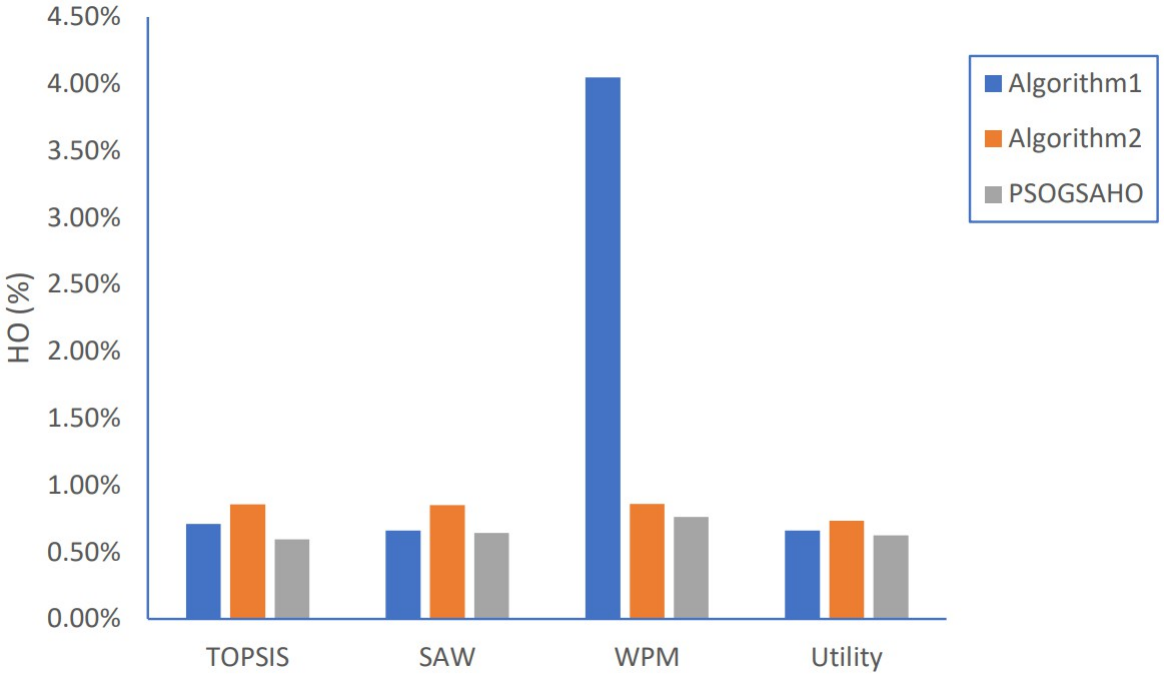
Handover percentage results.

**Fig 16:**
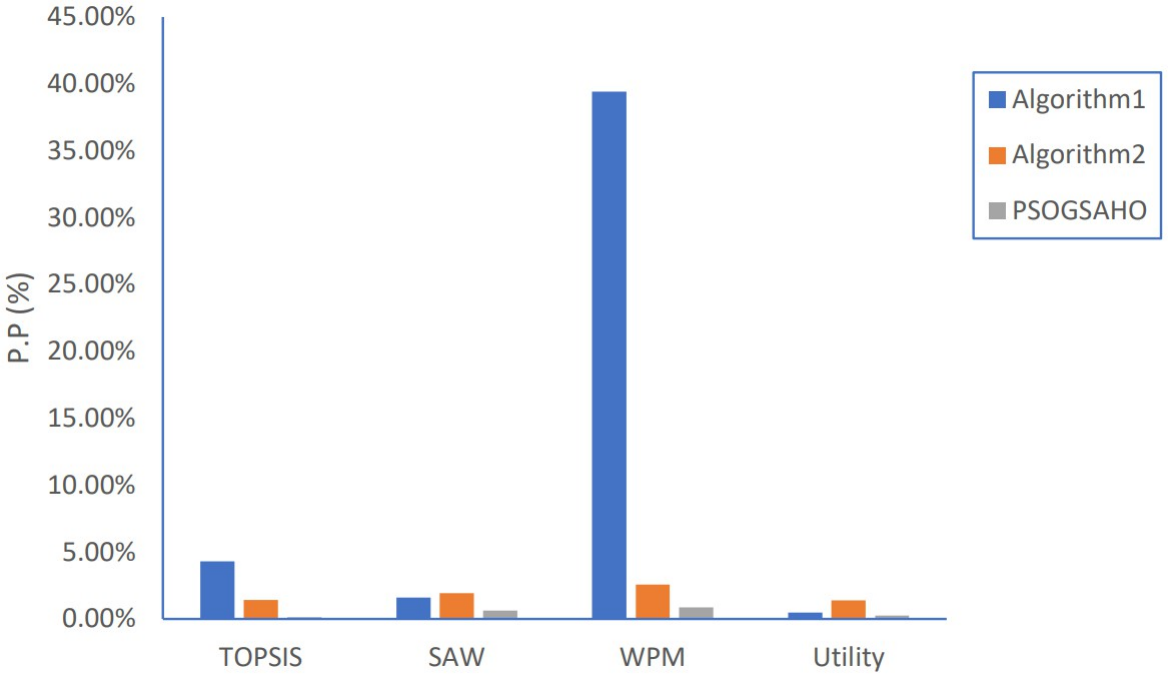
Ping-Pong percentage results.

**Fig 17:**
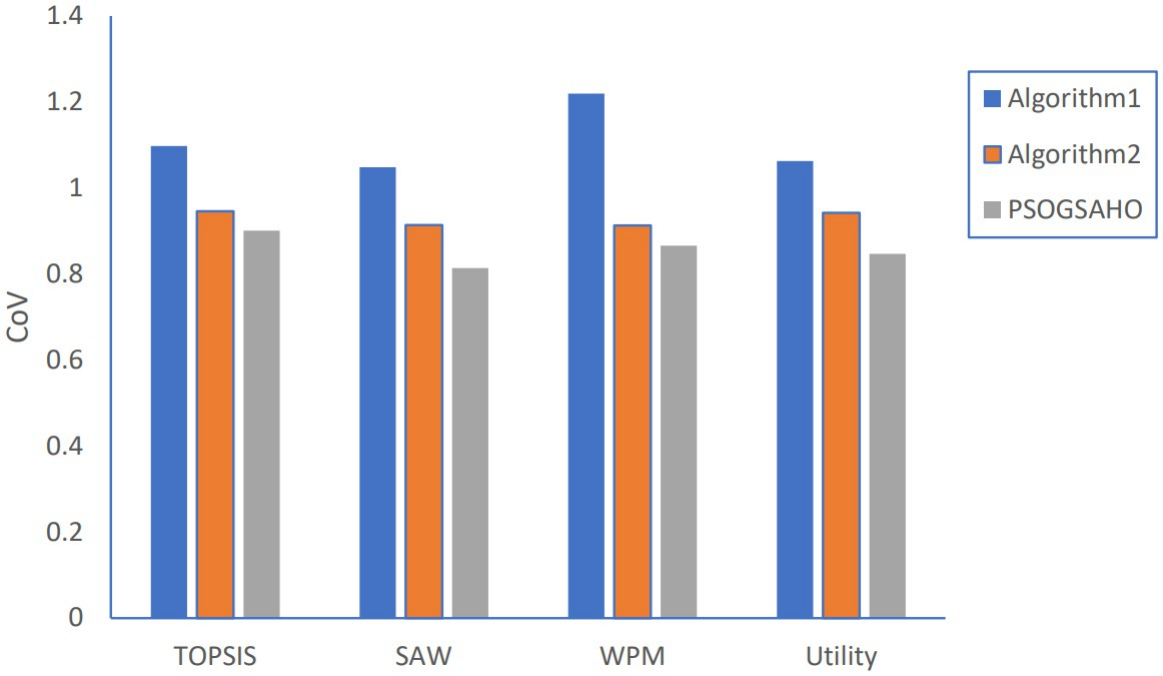
Load distribution results.

**Fig 18:**
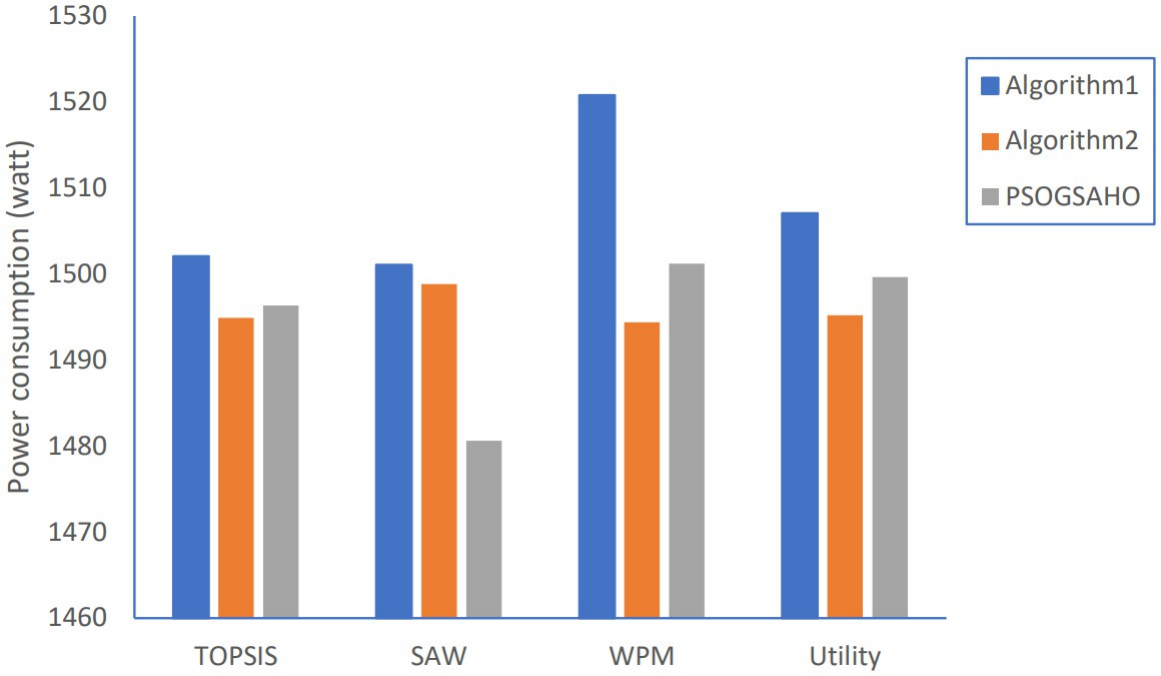
Power consumption results.

Next, the performance of the proposed PSOGSAHO algorithm is compared with the other algorithms, Algorithm 1 and Algorithm 2. Algorithm 2 is applied by assigning different values belonging to the defined range of both LF and SP and the average results are recorded. Regarding the PSOGSAHO algorithm, the optimization phase is performed to reach the optimum values of both LP and SP suitable for every strategy corresponding to the best performance. 50 iterations are used for the optimization procedure to guarantee that 10 candidate solutions for the search space will converge perfectly to the optimum solutions for the 2 parameters. The obtained optimized values of both LF and SP parameters are shown in Table 9. Comparative charts of the obtained results are shown in Figs 15–18.

**Table 9:** LF and SP optimum values using the PSOGSAHO algorithm.

Ranking Strategy	LF	SP
**TOPSIS**	0.35178	0.22972
**SAW**	0.34886	0.14862
**WPM**	0.35802	0.3
**Utility**	0.4286	0.12532

As shown in Fig 15 by introducing LF and SP parameters, Algorithm 2 has a little higher HO percentage in the case of TOPSIS, SAW, and Utility methods but manages to achieve a reduction of 70% for the WPM method. By applying the PSOGSAHO algorithm and using the obtained optimum values of LF and SP, the result for HO percentage is reduced for all strategies in ranges of 5% to 80% in the case of WPM, the most beneficiary strategy.

Regarding PP percentage, Algorithm 2 achieves better results for both TOPSIS and WPM methods while the PSOGSAHO algorithm succeeds in reducing unnecessary handovers to very small values whatever any strategy is applied especially the WPM strategy with PP percentage decreased by more than 97% compared to Algorithm 1.

For load distribution, as shown in Fig 17, by applying Algorithm 2; the CoV of HetNet load is reduced for all strategies by at least 12% to 25%. Better results are achieved with the PSOGSAHO algorithm by reducing the HetNet load CoV value by at least 4% to 11% compared to Algorithm 2. Also, for the amount of power consumption, the results show that Algorithm 2 and the PSOGSAHO algorithm perform better than Algorithm 1. The PSOGSAHO algorithm reduces the HetNet power consumption for all strategies by about 2% as shown in Fig 18. The lower the amount of power consumption, the better the performance of the HetNet as it will be able to serve more users for a longer duration, saving the network’s energy resources.

**Fig 19:**
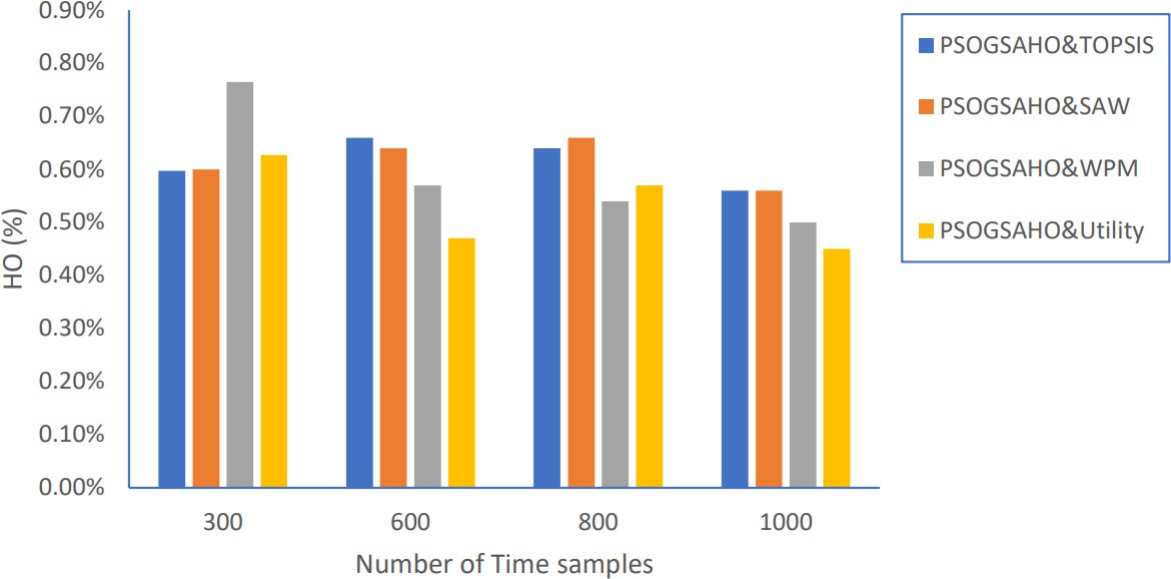
Handover percentage versus number of time samples.

Moreover, the performance of the proposed PSOGSAHO algorithm is evaluated by applying the obtained optimum solution by extending the number of time samples from 300 to 1000. As shown in Fig 19, the HO percentage is kept at an acceptable level and not increased by extending the time as a reflection of taking the handover decision efficiently.

**Fig 20:**
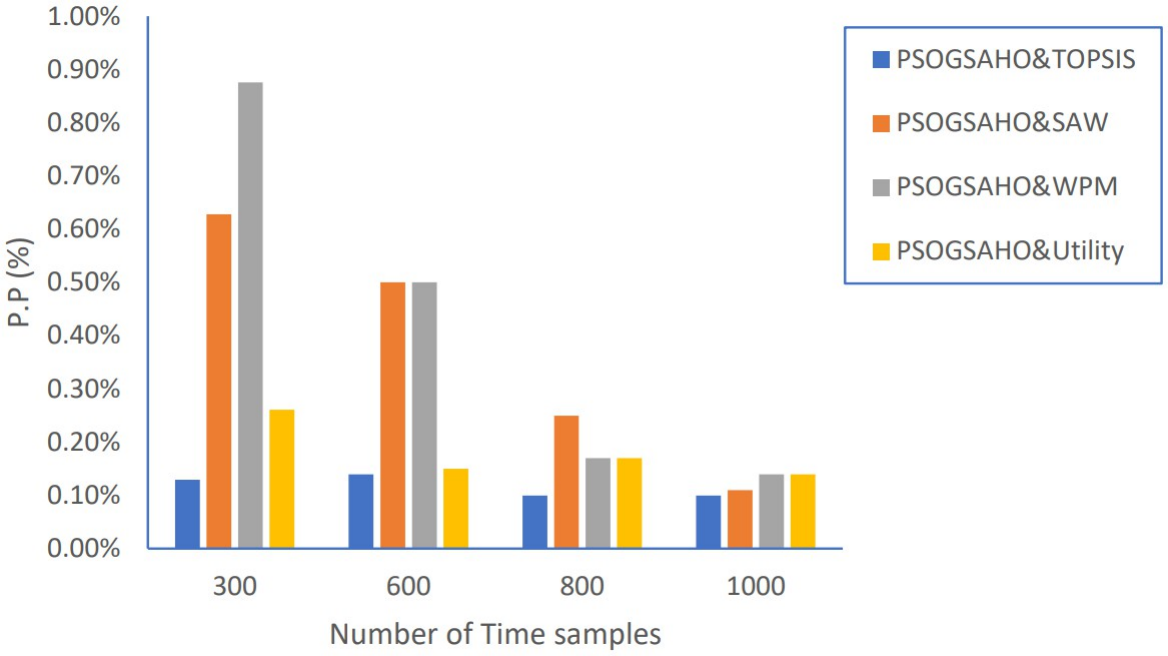
Ping-Pong percentage versus number of time samples.

**Fig 21:**
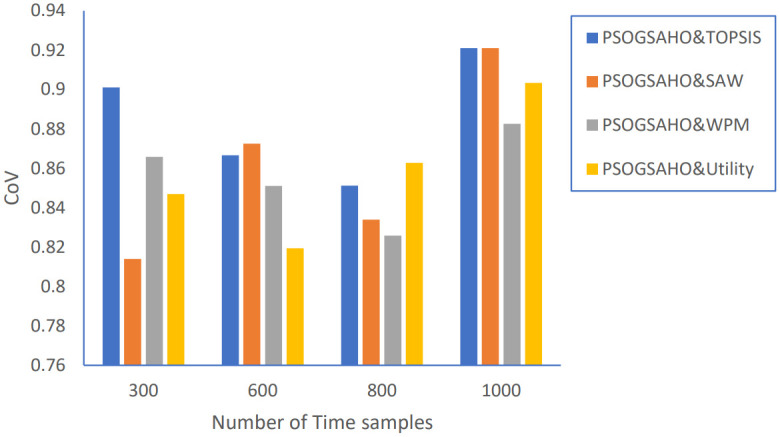
Load distribution versus number of time samples.

**Fig 22:**
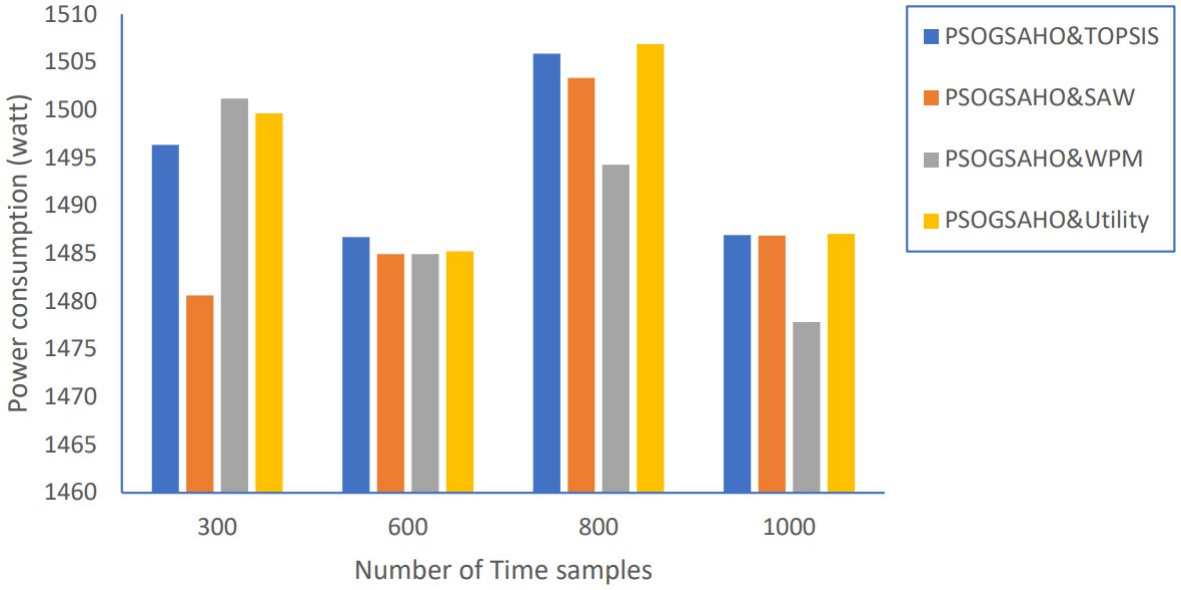
Power consumption versus number of time samples.

Regarding Ping-Pong percentage, as shown in Fig 20, the values are even decreased, which emphasizes achieving steady, efficient performance. Figs 21 and 22 show the load distribution and power consumption performance, respectively. Despite increasing the CoV values over time, the increasing percentage doesn’t exceed 10% for all strategies, which still outperforms the conventional algorithm represented earlier by Algorithm 0. Average power consumption values remain close to the initial optimum values within the range of 10 watts up and down, reflecting the proposed algorithm’s efficient behavior in distributing users over the HetNet.

**Fig 23:**
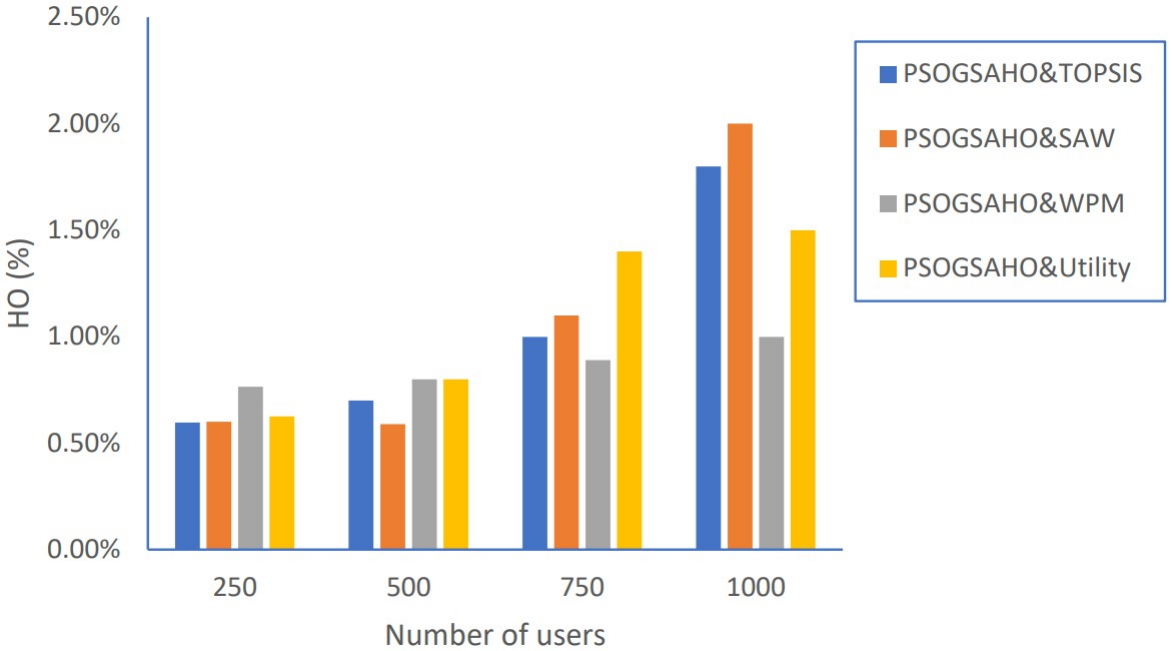
Handover percentage versus number of users.

**Fig 24:**
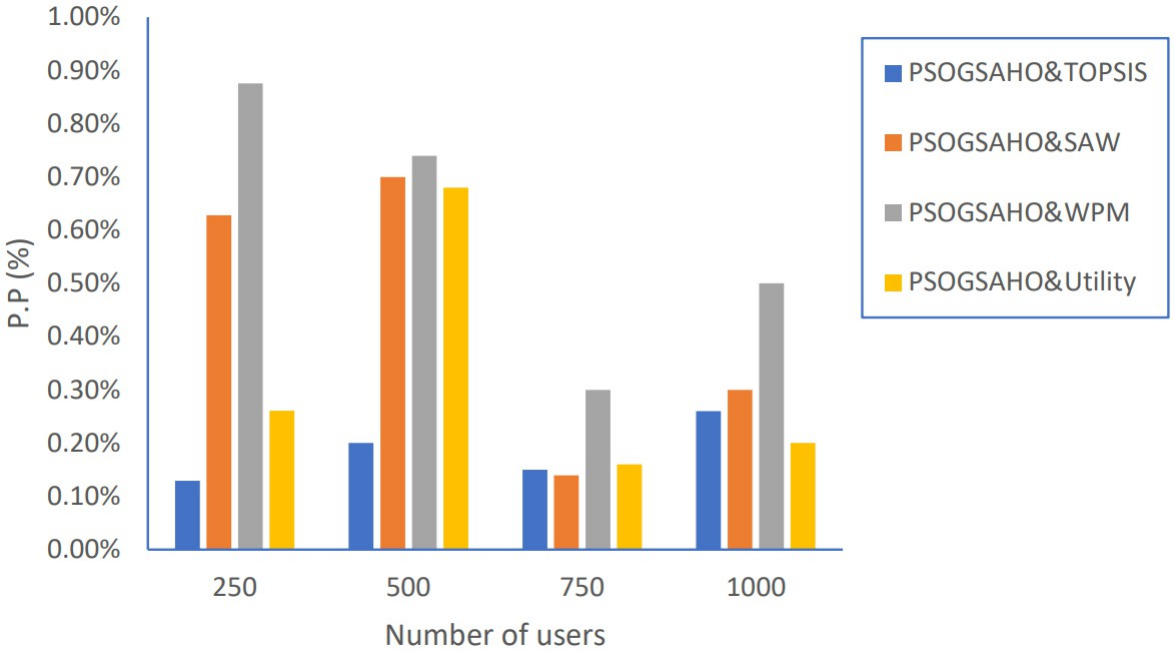
Ping-Pong percentage versus number of users.

**Fig 25:**
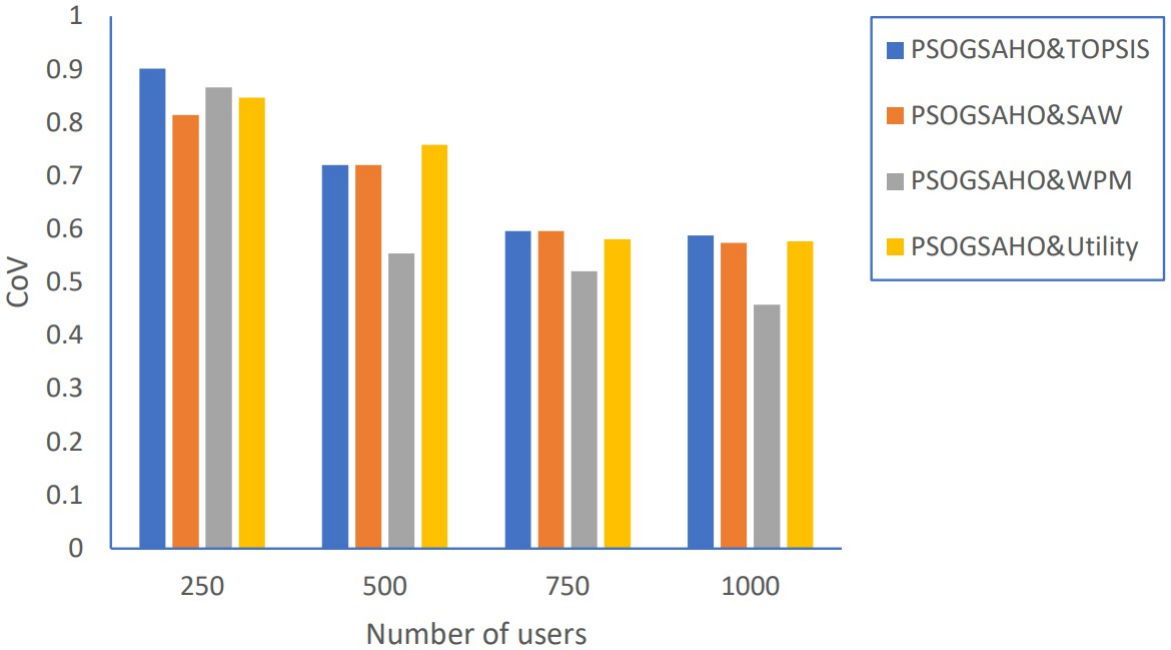
Load distribution versus number of users.

**Fig 26:**
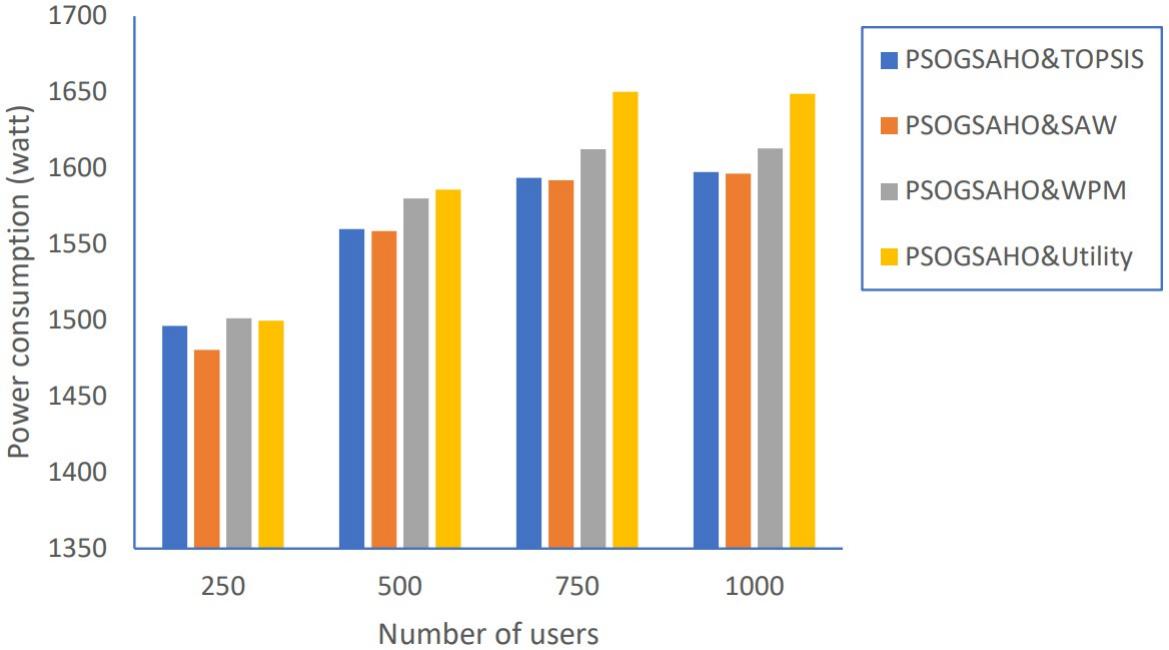
Power consumption versus number of users.

In addition, we extended the evaluation of the proposed PSOGSAHO algorithm in case of increasing the number of users as shown in Figs 23–26.

As shown in Fig 23, although a higher number of users yields to increase in the requested handovers; the proposed algorithm succeeded in containing the HO percentages within a 20% increasing range despite increasing the number of users by 4 times.

The proposed PSOGSAHO algorithm has remarkable performance regarding unnecessary handovers which remain at a low level and indeed decreased for most applied strategies even with duplicating the number of users twice as shown in Fig 24.

For the unbalanced load problem shown in Fig 25, the PSOGSAHO algorithm continues solving it and manages to distribute the increased load amount among all the involved HetNet networks. Regarding the amount of power consumption, the proposed algorithm utilizes all resources efficiently as with an increasing number of users 4 times; the average power consumption amount highest increased amount is only 10%, as shown in Fig 26.

## Conclusion and future work

Our paper introduces the PSOGSAHO algorithm, an automated vertical handover algorithm that adapts to changes in the working environment. It outperforms classic algorithms in selecting optimal networks for handover, considering user demands and network properties. The algorithm’s stability over time is advantageous, as it provides long-term optimal solutions, improving resource allocation and network management. The PSOGSAHO algorithm lays the foundation for future advancements and can be extended to prepare a dataset for efficient handover problem-solving using artificial intelligence systems. Overall, PSOGSAHO offers significant advantages for automated vertical handover.
